# 1033. Unexpected Allies: Vancomycin and Cefazolin Combination Therapy in Methicillin-Resistant *Staphylococcus aureus* Bacteremia

**DOI:** 10.1093/ofid/ofad500.064

**Published:** 2023-11-27

**Authors:** Kaylee E Caniff, Ashlan J Kunz Coyne, Michael J Rybak

**Affiliations:** Anti-Infective Research Lab, Eugene Applebaum College of Pharmacy and Health Sciences, Wayne State University, Royal Oak, MI; University of Kentucky, Detroit, Michigan; Eugene Applebaum College of Pharmacy and Health Sciences, Detroit, Michigan

## Abstract

**Background:**

Vancomycin (VAN) monotherapy is regarded as first-line treatment of bloodstream infections (BSIs) due to methicillin-resistant *Staphylococcus aureus* (MRSA). However, in vitro and clinical evidence indicate synergy is achieved against MRSA when certain beta-lactam antibiotics are combined with VAN. In 2016, a MRSA BSI treatment pathway was implemented at the Detroit Medical Center (DMC) which incorporates early use of combination therapy with VAN and cefazolin (CFZ). This study assesses clinical outcomes among patients receiving VAN vs. combination therapy with VAN + CFZ in MRSA BSI.

**Methods:**

This is a retrospective, observational cohort study of patients hospitalized with MRSA BSI at the DMC from 2006-2021. Included patients were initiated on VAN within 72 hours of the index culture. Patients were excluded if they received < 5 days of VAN, transferred to an outside hospital, received an alternative anti-MRSA antibiotic, received > 5 days of non-CFZ beta-lactam, or initiated on CFZ > 120 hours after index culture. Patients were compared by VAN therapy (no receipt of CFZ during treatment course) to VAN + CFZ combination therapy (CFZ initiated within 120 hours of index culture). Multivariable logistic regression was performed to identify independent predictors of 30-day mortality.

**Results:**

Overall, 359 patients were included; 145 received VAN + CFZ and 214 received VAN. There were no significant differences between groups when comparing baseline demographics, infectious source, comorbid conditions, MRSA risk factors, or illness severity. However, VAN + CFZ patients were more likely to have an infectious diseases consult (98.6% vs. 82.7%, p < 0.001). VAN + CFZ was associated a lower 30-day mortality rate (3.4% vs. 9.8%, p = 0.022) and shorter duration of bacteremia (49.0 [35.0-74.0] hours vs. 62.0 [42.0-95.8] hours, p = 0.005). After adjusting for confounders, VAN + CFZ receipt was independently associated with reduced 30-day mortality (aOR 0.31, 95% CI 0.11-0.93).

**Conclusion:**

In patients with MRSA BSI, the addition of CFZ to VAN within 120 hours of index culture was associated with improved outcomes. To our knowledge, this is the first study specifically examining VAN + CFZ in this population. Further research is warranted to determine the utility of this therapy combination in MRSA BSI.

**Disclosures:**

**Michael J. Rybak, PharmD, PhD, MPH**, Abbvie, Merck, Paratek, Shionogi, Entasis, La Jolla, T2 Biosystems: Advisor/Consultant

